# Incidence and outcomes of delayed presentation and surgery in peritoneal surface malignancies

**DOI:** 10.3389/fonc.2023.1137785

**Published:** 2023-05-30

**Authors:** Jun Kiat Thaddaeus Tan, Jolene Si Min Wong, Chin Jin Seo, Cindy Lim, Hong-Yuan Zhu, Chin-Ann Johnny Ong, Claramae Shulyn Chia

**Affiliations:** ^1^ Department of Sarcoma, Peritoneal and Rare Tumors (SPRinT), Division of Surgery and Surgical Oncology, National Cancer Centre Singapore, 11 Hospital Crescent, Singapore, Singapore; ^2^ Department of Sarcoma, Peritoneal and Rare Tumors (SPRinT), Division of Surgery and Surgical Oncology, Singapore General Hospital, Singapore, Singapore; ^3^ SingHealth Duke-NUS Oncology Academic Clinical Program, Duke-NUS Medical School, Singapore, Singapore; ^4^ SingHealth Duke-NUS Surgery Academic Clinical Program, Duke-NUS Medical School, Singapore, Singapore; ^5^ Division of Clinical Trials and Epidemiological Sciences, National Cancer Centre Singapore, Singapore, Singapore; ^6^ Laboratory of Applied Human Genetics, Division of Medical Sciences, National Cancer Centre Singapore, 11 Hospital Crescent, Singapore, Singapore; ^7^ Institute of Molecular and Cell Biology, A*STAR Research Entitie, Singapore, Singapore

**Keywords:** peritoneal malignancy, cytoreductive surgery, hyperthermic intraperitoneal chemotherapy, delay, survival

## Abstract

**Background:**

Peritoneal surface malignancies (PSM) present insidiously and often pose diagnostic challenges. There is a paucity of literature quantifying the frequency and extent of therapeutic delays in PSM and its impact on oncological outcomes.

**Methods:**

A review of a prospectively maintained registry of PSM patients undergoing Cytoreductive Surgery and Hyperthermic Intra-peritoneal Chemotherapy (CRS-HIPEC) was conducted. Causes for treatment delays were identified. We evaluate the impact of delayed presentation and treatment delays on oncological outcomes using Cox proportional hazards models.

**Results:**

319 patients underwent CRS-HIPEC over a 6-years duration. 58 patients were eventually included in this study. Mean duration between symptom onset and CRS-HIPEC was 186.0 ± 37.1 days (range 18-1494 days) and mean duration of between patient-reported symptom onset and initial presentation was 56.7 ± 16.8 days. Delayed presentation (> 60 days between symptom onset and presentation) was seen in 20.7% (n=12) of patients and 50.0% (n=29) experienced a significant treatment delay of > 90 days between 1^st^ presentation and CRS-HIPEC. Common causes for treatment delays were healthcare provider-related i.e. delayed or inappropriate referrals (43.1%) and delayed presentation to care (31.0%). Delayed presentation was a significantly associated with poorer disease free survival (DFS) (HR 4.67, 95% CI 1.11-19.69, p=0.036).

**Conclusion:**

Delayed presentation and treatment delays are common and may have an impact on oncological outcomes. There is an urgent need to improve patient education and streamline healthcare delivery processes in the management of PSM.

## 1 Introduction

Patients with peritoneal surface malignancies (PSM) represent a heterogenous group ranging from primary peritoneal cancer to peritoneal metastases secondary to various primaries. Since its introduction in the 1980s, Cytoreductive Surgery and Hyperthermic Intraperitoneal Chemotherapy (CRS-HIPEC) has revolutionized the management of peritoneal malignancies (1, 2). When once associated with a dismal prognosis, selected patients receiving optimal treatment now boost a 10 year survival, with rates of up to 63% reported in patients with pseudomyxoma peritonei (1, 3–6). CRS-HIPEC is now widely regarded as central to the management of PSM in selected patients (6–8).

However, PSM are frequently clinically occult with patients presenting insidiously and with nonspecific symptoms thereby posing significant diagnostic challenges (9–16). Establishing a histological diagnosis often proves challenging, and even at the tertiary level, interpretation of potentially indeterminate imaging characteristics and difficulties in determining histological characteristics result in further delay towards timely diagnosis and treatment of such malignancies (9, 17). In addition, general awareness, and knowledge amongst the physician population towards PSM is poor. In a locally conducted survey, up to 50% of survey participants acknowledged that they were unfamiliar with the disease entity and were unaware of the presence of local PSM specialist units for referrals (18), representing a potential source contributing to delayed specialist review.

Several studies suggest a significant correlation between delays incurred in diagnostic evaluation and poorer oncologic outcomes in various tumor histologies (19–25). While the current evidence in literature concurs that delivery of curative surgery in an expedient manner is crucial to the optimal management of PSM (19, 26), the causative factors and overall impact of delays incurred from patient and healthcare-related factors on oncologic outcomes has not been well-studied.

Therefore, we aim to evaluate the incidence and causes of delayed presentation and surgery and examine its impact on oncological outcomes in patients with PSM.

## 2 Materials and methods

### 2.1 Patient selection and data

The study was performed in a single tertiary institution. Data was retrieved from a prospectively maintained database of patients treated with CRS-HIPEC for PSM between January 2014 and September 2019. The study was conducted with the approval of the Centralized Institutional Review Board (CIRB) of Singapore Health Services, CIRB reference number 2018/2638.

We included patients undergoing their index CRS-HIPEC surgery after a primary diagnosis of PSM. Patients with (i) recurrent PSM on a background of previously treated peritoneal malignancy, (ii) peritoneal metastases of a previously known and treated primary tumor, or (iii) who underwent neoadjuvant chemotherapy prior to CRS-HIPEC, were excluded.

**Figure 1:**
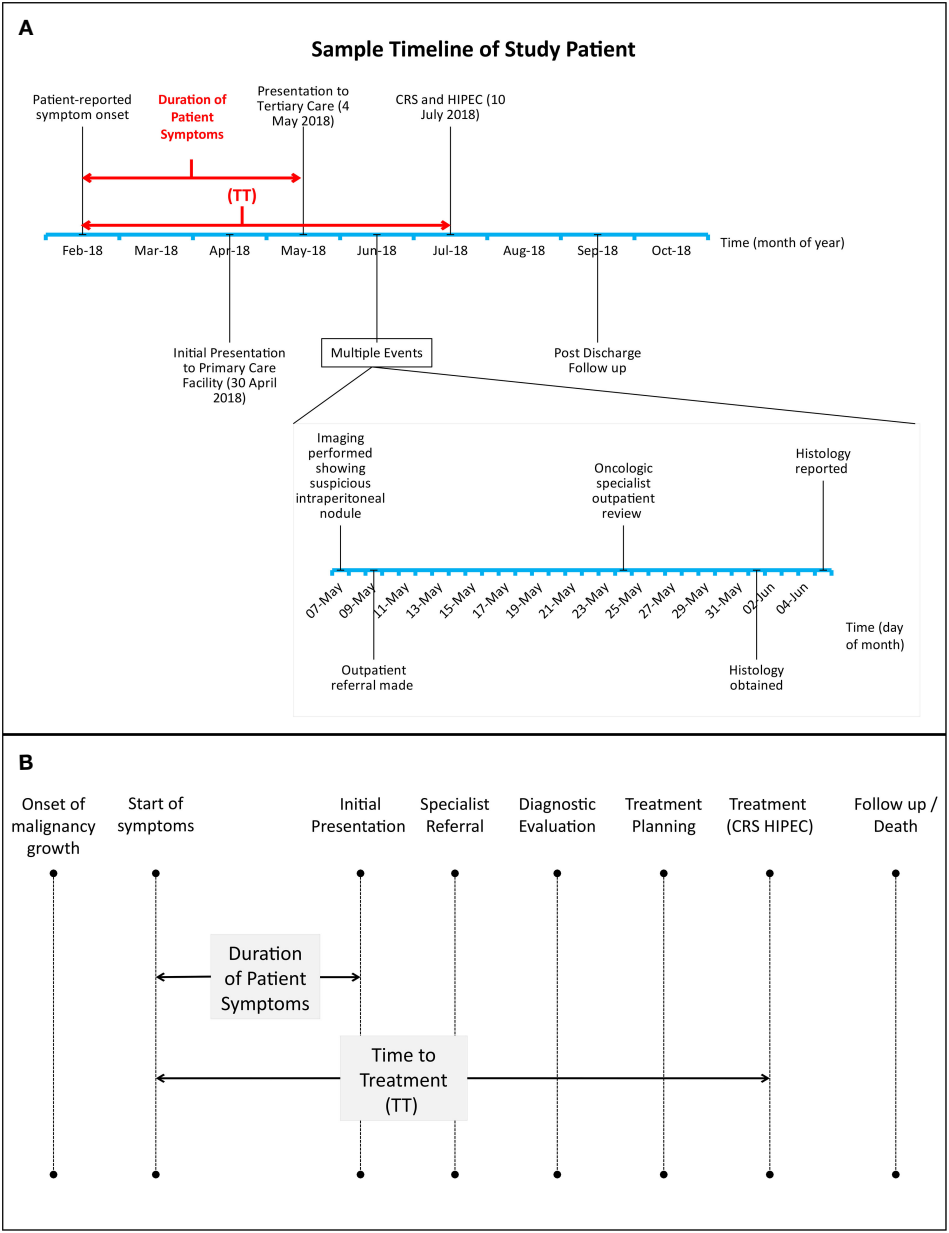
**(A, B)**: **(A)**, sample timeline of study patient treated for high grade serous carcinoma of the fallopian tubes with peritoneal involvement. Dates of initial symptom onset, presentation to tertiary care, further diagnostic evaluation and CRS-HIPEC were recorded for every patient. CRS-HIPEC=Cytoreductive surgery, hyperthermic intraperitoneal chemotherapy. TT=time to treatment. **(B)**, Relationship between duration of patient symptoms and time to treatment (TT) with respect to key time points in cancer-related treatment delays. Adapted from *Mou et al.* (20).

Data on patient demographics, onset and duration of symptoms attributable to their primary malignancy, preoperative clinical course and oncologic history was obtained *via* a thorough retrospective evaluation of prospectively maintained clinical records. Patients with insufficient data pertaining to symptoms prior to initial presentation on clinical records were excluded from this study. Descriptive analyses were performed on these variables and survival outcomes were evaluated. A virtual diagnostic and treatment timeline was generated for each study patient and contributory factors to treatment delays were identified by the authors on a case-by-case basis and analyzed (**Figures 1A, B**).

### 2.2 Key definitions

1. Delayed Presentation: We defined this as a duration of > 60 days between patient-reported symptom onset and 1^st^ presentation at a healthcare institution.
2. Time to Treatment (TT): Duration between patient-reported symptom onset and CRS-HIPEC surgery.
3. Delayed TT: We defined this as a Time to Treatment of > 90 days.

Given the paucity of evidence for the expected treatment timelines in peritoneal malignancies in the current literature base, the cutoffs for delayed presentation and delayed TT of 60 and 90 days, respectively, were set arbitrarily on agreement with all authors based on our institutional experience with PSM and its expected treatment course.

### 2.3 Factors contributing to delays

Factors contributing to delays of any duration were identified on a case-by-case basis and classified into 5 categories: Delayed presentation, healthcare provider, healthcare system, disease or patient-related (**Table 1**).

**Table 1:** Factors contributing to Delayed Time to Treatment.

Factors	Explanation	Examples of Inclusions
**Delayed Presentation**	This is patient-driven and a result of lack of awareness and knowledge of symptoms and failure to seek appropriate medical services after onset of symptoms.	* Outright dismissal of symptoms * Perception of symptoms as mild and not warranting medical attention
**Healthcare Provider**	This is driven by the lack of awareness and knowledge of primary and tertiary healthcare providers such that a prompt referral to a peritoneal surgical specialist was delayed	* Elective referrals from external institutions * Elective referrals from primary care providers * Referrals made to disciplines without specialized surgical capabilities
**Healthcare System**	This is due to the lack of hospital-based resources e.g. operating theatre slots, long patient-waiting time prior to specialist review	* Delayed surgical case listing due to scheduling conflicts * Prolonged interval of follow up due to difficulty obtaining appointment slot
**Disease-related**	This is due to any disease-related complications or diagnostic difficulties encountered after presentation to a peritoneal specialist and is due to the nature of peritoneal disease and non-diagnostic findings based on radiological or histological investigations.	* Delayed reporting of diagnostic radiological or histology findings * Prolonged course of treatment planning including delays incurred by listing for and discussion at multidisciplinary team conferences * Indeterminate initial findings warranting serial monitoring for disease manifestation or progression resulting in delays * Delays incurred from an evolving disease morphology causing change in treatment plan
**Patient-related**	This is delay because of patient factors such as other co-morbidities that require optimization, defaulted visits or follow-up, refusal to undergo prompt surgical intervention despite medical advice.	* Treatment of other nonrelated illness * Missed follow ups due to intercurrent nonrelated illnesses * Delays incurred from initial refusal of surgery

### 2.4 CRS and HIPEC

CRS and HIPEC performed at our institution included resection of the primary tumor with resection of all macroscopic peritoneal deposits combining peritonectomy procedures as well as resection of any involved intraabdominal visceral organs to achieve complete cytoreduction, with subsequent administration of HIPEC. HIPEC was performed in a closed technique, with administration of mitomycin C at 41-42°C over a duration of 60 minutes.

### 2.5 Statistical methods and survival analysis

Differences in characteristics were tested using the Wilcoxon rank sum test for continuous variables or Fisher’s exact test for categorical variables. Kaplan-Meier survival functions were used to analyze the impact of treatment delays with respect to time to treatment and symptom duration. Overall survival (OS) was defined as time from CRS-HIPEC to death from all causes or censored at last follow-up. Disease-free survival (DFS) was defined as time from CRS-HIPEC to disease progression or censored at death or last follow-up. The Cox proportional hazards model was used to model association between survival endpoints and patient characteristics, adjusted for tumor histology. Differences between groups were estimated using the log-rank test. A two-sided p-value of less than 0.05 was considered statistically significant. All analyses were performed in R software (version 4.2.0).

## 3 Results

### 3.1 Overall characteristics

319 patients underwent CRS-HIPEC at the National Cancer Centre Singapore and Singapore General Hospital between January 2014 and September 2019. 60% (n=194) underwent surgery for recurrent peritoneal disease or PM arising from a previously known and treated primary tumor and were excluded from the study. 6.3% (n=20) had insufficient data on preoperative presentation and were excluded. A further 14.7% (n=47) underwent neoadjuvant chemotherapy prior to definitive surgery, and were excluded. Finally, 58 patients were included into this study. Patients were followed up for an average of 12.4 months from time of surgery. A summary of demographic and clinical characteristics of patients studied is listed in **Table 2**.

**Table 2:** Demographics and clinical characteristics.

	All patients (n=58)	No Delay in TT (≤90days) (n=29)	Delayed TT (>90days) (n=29)	p-value
Age at CRS-HIPEC, years				0.963
Mean(SD)	57.2 (10.8)	57.1 (12.1)	57.3 (9.7)	
Median (IQR)	59.0 (50.9, 64.4)	59.4 (46.9, 64.4)	58.9 (52.1, 66.3)	
Range	32.0 - 77.7	32.8 - 77.7	32.0 - 72.7	
Sex				0.263
Female	39 (67.2)	22 (75.9)	17 (58.6)	
Male	19 (32.8)	7 (24.1)	12 (41.4)	
Ethnicity				0.474
Chinese	42 (72.4)	23 (79.3)	19 (65.5)	
Indian	5 (8.6)	3 (10.3)	2 (6.9)	
Malay	3 (5.2)	1 (3.4)	2 (6.9)	
Others	8 (13.8)	2 (6.9)	6 (20.7)	
ECOG performance status				0.894
1	44 (75.9)	21 (72.4)	23 (79.3)	
2	4 (6.9)	2 (6.9)	2 (6.9)	
Unspecified	10 (17.2)	6 (20.7)	4 (13.8)	
Histology				0.338
LAMN/HAMN	29 (50.0)	13 (44.8)	16 (55.2)	
PMCA	15 (25.9)	10 (34.5)	5 (17.2)	
Primary peritoneal	14 (24.1)	6 (20.7)	8 (27.6)	
PCI score				0.106
Mean(SD)	14.7 (11.0)	12.2 (10.2)	17.1 (11.4)	
Median (IQR)	15.0 (3.5, 24.0)	13.0 (2.0, 17.0)	17.0 (6.2, 27.8)	
Range	0.0 - 32.0	0.0 - 31.0	0.0 - 32.0	
Unspecified	7	4	3	
CC score				0.941
0 (No tumour)	35 (60.3)	18 (62.1)	17 (58.6)	
1 (<2.5mm)	10 (17.2)	5 (17.2)	5 (17.2)	
2 (2.5mm - 2.5cm)	3 (5.2)	2 (6.9)	1 (3.4)	
3 (> 2.5cm)	2 (3.4)	1 (3.4)	1 (3.4)	
Unspecified	8 (13.8)	3 (10.3)	5 (17.2)	
Comorbidities				
Hypertension	18 (31.0)	12 (41.4)	6 (20.7)	0.155
Diabetes	7 (12.1)	5 (17.2)	2 (6.9)	0.423
Hyperlipidemia	13 (22.4)	10 (34.5)	3 (10.3)	0.056
IHD	3 (5.2)	1 (3.4)	2 (6.9)	1.000
COPD	0 (0.0)	0 (0.0)	0 (0.0)	NA
Asthma	0 (0.0)	0 (0.0)	0 (0.0)	NA
Other malignancies	2 (3.4)	2 (6.9)	0 (0.0)	0.491
Others	31 (53.4)	18 (62.1)	13 (44.8)	0.292
None	10 (17.2)	4 (13.8)	6 (20.7)	0.730

Data presented as No. (%) unless otherwise indicated. CC, completion of cytoreduction score; COPD, Chronic Obstructive Pulmonary Disease; CRS-HIPEC, Cytoreductive Surgery and Hyperthermic Intraperitoneal Chemotherapy; GI, Gastrointestinal; HAMN, High Grade Appendiceal Mucinous Neoplasm; IHD, ischemic heart disease; IQR, interquartile range; LAMN, Low Grade Appendiceal Mucinous Neoplasm; PCI, Peritoneal Carcinomatosis Index; PMCA, Peritoneal Mucinous Carcinomatosis; SD, standard deviation; TT, Time to Treatment; NA, not applicable. NA, not applicable.

### 3.2 Patient symptoms, incidence of delayed treatment and primary contributing factors

Common presenting complaints were varied and include abdominal discomfort, distension and constitutional symptoms including loss of weight and appetite. Patients who were asymptomatic or had extra-abdominal symptoms not attributable to PSM were assigned a symptom duration of zero days. The mean duration between patient-reported symptom onset and CRS-HIPEC (TT) was 186.0 ± 37.1 days (range 18-1494 days) and mean duration of between patient-reported symptom onset and initial presentation to any healthcare institution was 56.7 days (SD ± 16.8, range 0-730). 29(50.0%) experienced prolonged TT of more than 90 days; while 12(20.7%) patients were found to have a delayed presentation of more than 60 days. Among patients included into this study, healthcare-provider related delays (43.1%), delayed presentation (31.0%) were identified as the predominant causes of delayed TT. Among the patients who suffered healthcare provider related delays, 17(68.0%) patients were first evaluated in centers which were not specialized in treatment of peritoneal malignancies, and 6(24.0%) incurred delays after being inappropriately referred from primary care providers to disciplines not equipped to manage peritoneal disease. Patients who encountered treatment delays attributable to delayed presentation predominantly experienced protracted symptoms prior to making a decision to seek medical attention.

**Figure 2:**
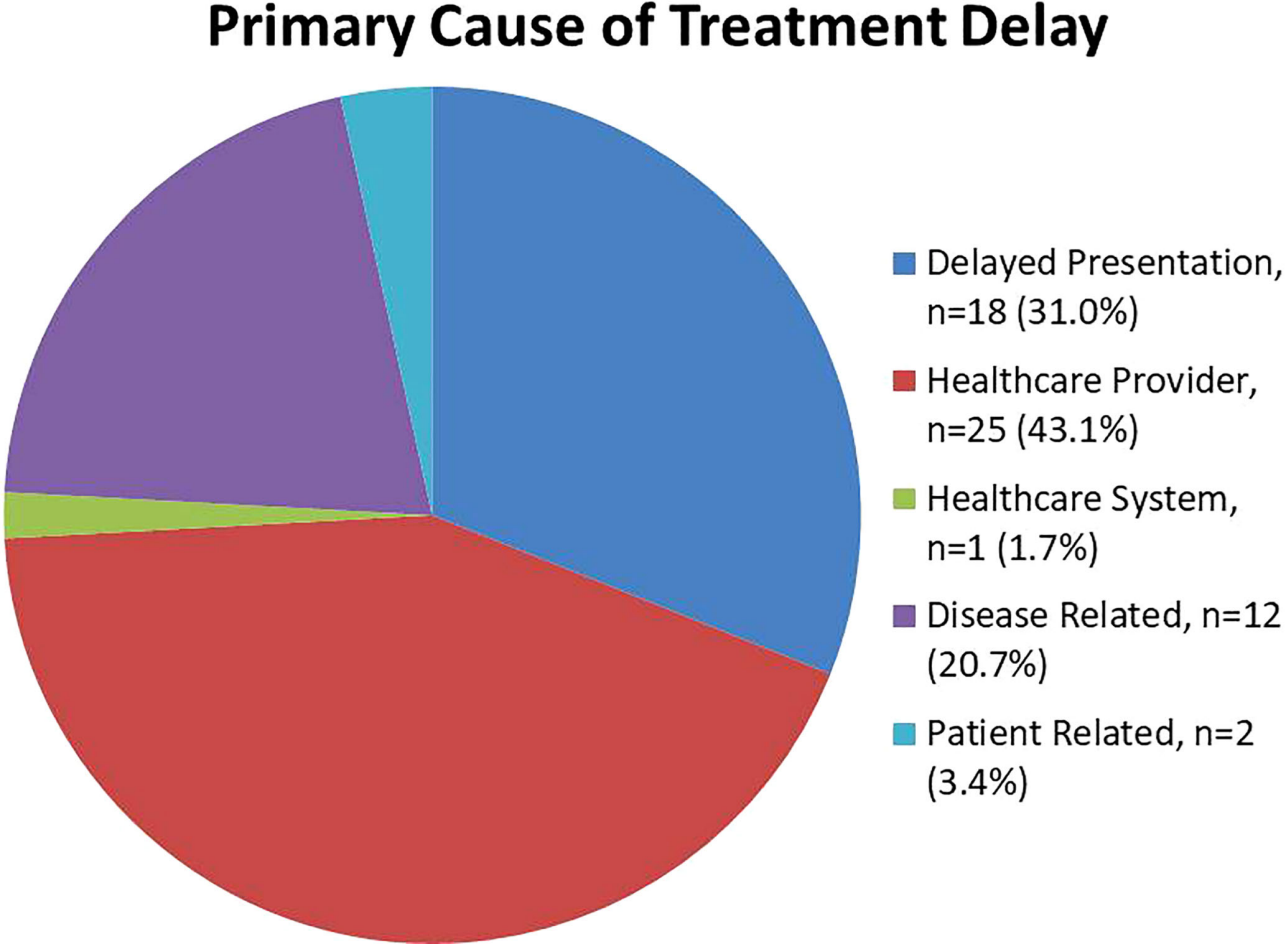
Analysis of factors contributing to treatment delays in peritoneal malignancies.

Less common causes for treatment delays were disease related issues (20.7%), patient related (3.4%) and healthcare system related delays (1.7%) (**Figure 2**). A comprehensive list of factors included under each category is shown in **Table 3**.

**Table 3:** Cox regression analysis of disease-free survival and overall survival by demographic and clinical variables.

	Disease-free survival			Overall survival		
	E/N	HR (95%CI)	p-value	E/N	HR (95%CI)	p-value
Delayed Presentation						
No delay (≤60 days)	5/46	1		3/46	1	
Delayed (>60 days)	4/12	3.65 (0.91-14.62)	0.0677	3/12	9.93 (1.03-95.89)	0.0473
Time to Treatment (TT)						
No delay (≤90 days)	4/29	1		3/29	1	
Delayed (>90 days)	5/29	1.04 (0.27-3.93)	0.9587	3/29	1.91 (0.30-12.13)	0.4913
Age at CRS-HIPEC, years						
<60	4/32	1		3/32	1	
≥ 60	5/26	3.76 (0.92-15.30)	0.0644	3/26	5.39 (0.84-34.72)	0.0764
Sex						
Female	4/39	1		3/39	1	
Male	5/19	2.85 (0.68-11.96)	0.1519	3/19	5.64 (0.55-58.03)	0.1457
ECOG performance status						
1	8/44	1		5/44	1	
2	1/4	1.30 (0.15-11.72)	0.8129	1/4	1.74 (0.18-17.11)	0.6364
Unspecified	0/10	Not estimable		0/10	Not estimable	
Histology						
PMCA	5/15	1		4/15	1	
LAMN/HAMN	1/29	0.12 (0.01-1.06)	0.0569	0/29	Not estimable	
Primary peritoneal	3/14	1.19 (0.27-5.34)	0.8202	2/14	2.18 (0.29-16.39)	0.4506

E/N stands for Event/Number, in the case of OS, event is the number of death, in the case of DFS, event could be relapse or death. CI, Confidence interval, CRS-HIPEC, Cytoreductive Surgery and Hyperthermic Intraperitoneal Chemotherapy; GI, Gastrointestinal; HAMN, High Grade Appendiceal Mucinous Neoplasm, LAMN, Low Grade Appendiceal Mucinous Neoplasm, PMCA, Peritoneal Mucinous Carcinomatosis.

Two patients were excluded from disease-free survival as they were lost to follow-up after CRS-HIPEC.

### 3.3 Relationship between delayed presentation, time to treatment and survival

**Figure 3:**
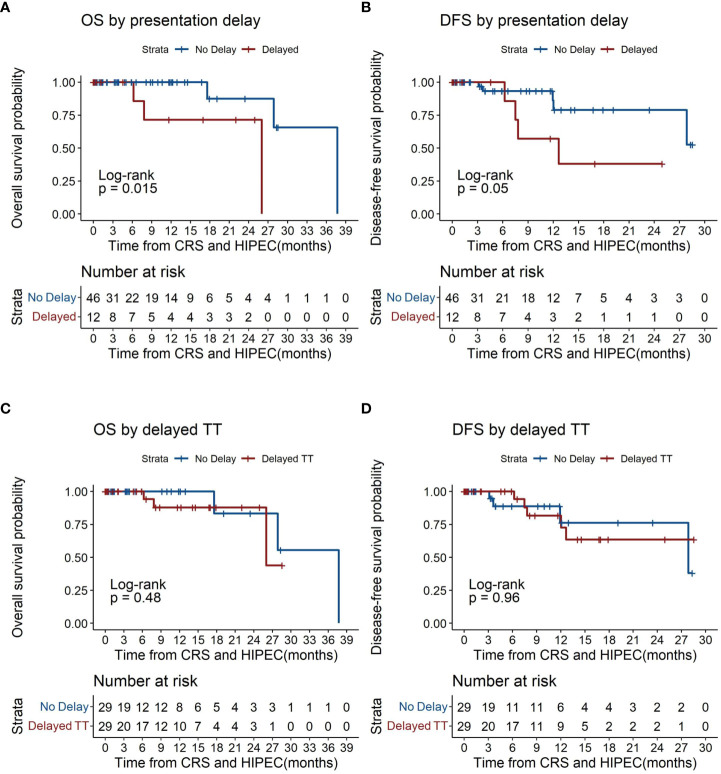
Kaplan-Meier plots of overall survival and disease-free survival by time to treatment (TT). **(A)** overall survival by presentation delay. **(B)** disease free survival by presentation delay. **(C)** overall survival by time to treatment. **(D)** disease free survival by time to treatment. CRS, Cytoreductive Surgery; HIPEC, Hyperthermic intraperitoneal chemotherapy; TT, time to treatment.

Patients with delayed presentation demonstrated poorer overall survival compared to those without (p=0.015, **Figure 3A**), with median overall survival (OS) being 26.0 months for patients with delayed presentation. 1- and 2- year OS was 100% (95% CI 100-100) and 71.4% (95% CI 44.7-100) versus 100% and 100% respectively for patients with and without delayed presentation. Delayed patient presentation was associated with poorer survival (HR 9.93, 95% CI 1.03-95.89, p=0.047), although this was non-significant after adjustment for tumor histology (HR 7.91, 95% CI 0.72-87.15, p=0.091).

Patients with delayed presentation similarly demonstrated lower rates of DFS (p=0.05, **Figure 3B**) with median disease free survival (DFS) at 12.7 months for patients with delayed presentation. 1- and 2- year DFS was 100% and 57.1% (95% CI 30.1-100) versus 93.3% (95% CI 84.8-100) and 86.1% (95% CI 71.7-100) respectively for patients with and without delayed presentation. Patients with delayed presentation demonstrated a trend towards poorer DFS (HR 3.65, 95% CI 0.91-14.62, p=0.068) which was significant after correcting for tumor histology (HR 4.67, 95% CI 1.11-19.69, p=0.036) (**Table 3**).

Overall survival was similar between patients with and without delayed TT (p=0.48, **Figure 3C**). Among patients with delayed TT, median overall survival (OS) was 26.0 months. 1- and 2- year OS was 100% and 87.8% (95% CI 73.4-100) versus 100% and 100% respectively for those with and without delayed TT. No statistical difference in overall survival was demonstrated between patients with and without delayed TT (HR 1.91, 95% CI 0.30-12.13, p=0.491) (**Table 3**).

No significant differences were found in disease free survival between patients with and without delayed TT (p=0.96, **Figure 3D**). Among patients with delayed TT, median DFS was 4 months. 1- and 2- year DFS was 100% and 81.6% (95% CI 64.7-100) versus 100% and 100% respectively for those with and without delayed TT (**Table 3**).

In view of the limited number of events and small sample size, 95% confidence intervals for median OS and DFS for patients with and without delayed TT, and similarly for those with and without presentation delay, could not be estimated.

## 4 Discussion

Our study demonstrates insight into the factors contributing to treatment delays in PSM, which consist of several potentially actionable causes predominantly attributable to provider-related delays incurred in work processes involved in transfer of care between healthcare institutions (43.1%). These findings represent, to our knowledge, the first attempt in current literature at describing actionable factors contributing to treatment delays in peritoneal malignancies. Our findings also suggest that delayed presentation may result in poorer overall survival and disease-free survival among patients with PSM. Interestingly, however, a delayed time to treatment of >90 days did not result in any significant difference in overall survival or disease-free survival in our study cohort. While the literature consistently supports the early diagnosis and treatment of peritoneal malignancies (27–29) the quantitative effect of diagnostic and treatment delays on oncologic outcomes in such patients has not been sufficiently studied. Furthermore, given the highly litigated nature of oncologic practice in general, delayed evaluation and treatment of such conditions may prove also to be a significant source of financial and legal burden to healthcare systems worldwide (30). Therefore, quantifying the causative factors of treatment delays and identifying the impact of such treatment delays on oncologic outcomes is crucial to addressing public health concerns pertaining to the evaluation and treatment of peritoneal malignancies.

A large proportion of study patients suffered delays attributable to provider-related delays incurred by processes involved in transfer of care between institutions (43.1%) and delayed presentation (31.0%) predominantly contributed by a lack of patient awareness regarding signs and symptoms of peritoneal disease. These findings suggest that treatment delays are predominantly influenced by factors within the healthcare system that can be further mitigated with improved professional education and streamlining of administrative processes. Considering the well-documented challenges involved in evaluation of peritoneal malignancies given their significant heterogeneity, varying nature of initial presentation and overall rare occurrence (14, 15, 17, 31–34), healthcare professionals at large would benefit from better awareness on the initial presentations, appropriate evaluation strategies and treatment options for peritoneal malignancies. Additionally, treatment delays incurred here are also contributed at least in part by patients’ failure to recognize symptoms that suggest underlying peritoneal malignancies which resulted in delayed presentation to primary care. Findings from our study similarly highlight the importance of early patient recognition of symptoms and early appropriate clinical evaluation in optimizing treatment outcomes for peritoneal surface malignancies.

This study’s findings on the association between delayed presentation and poorer overall survival and disease-free survival must be interpreted with due consideration given to the limited sample size and relatively short follow up duration of 12.4 months post CRS-HIPEC, also taking into consideration the effects of disease biology on symptom progression and presentation. The detrimental effect of delayed presentation on survival outcomes may potentially be attributable to poorer disease biology, with more indolent biology presenting with more clinically occult symptoms (28, 29) which translates to a delayed presentation to medical attention, and ultimately poorer survival outcomes. However, our study notably demonstrated that tumor histology and patient comorbidities appear relatively consistent in both non-delayed and delayed time to treatment groups (**Table 2**), suggesting that such factors were not likely to have influenced the duration of time to treatment. This study further demonstrated an independent association between delayed presentation and reduced disease free survival which persisted after correction for tumor histology (HR 4.67, 95% CI 1.11-19.69, p=0.036), which suggests that delayed presentation of more than 60 days may adversely impact disease outcomes in PSM regardless of the contributory primary histology. The persistent correlation between delayed presentation and poorer survival outcomes after correction for histology would, however, suggest that delayed presentation results in poorer survival due to other independent factors, which has been similarly demonstrated for some other biologically distinct malignancies, delayed presentation in soft tissue sarcoma for instance having demonstrated to be associated with higher likelihood of distal metastases on diagnosis, portending poorer prognoses and poorer survival outcomes (23).

While age does not appear to significantly influence survival outcomes in patients with PM based on our data, older patients 60 years of age and above with peritoneal malignancies demonstrate a tendency towards poorer survival (HR 5.39, 95% CI 0.84-34.72, p=0.076) and lower DFS (HR 3.76, 95% CI 0.92-15.30) compared to younger patients (**Table 3**). This emphasizes the importance of early detection of PM in particular for older patients, although more studies with a longer follow up duration would be required to prove a significant correlation between age and oncological outcomes in PM.

This study bears several other limitations. Firstly, data collected on pre-hospital symptoms relies on patient-reported duration and severity of symptoms which may bear an inherent recall bias at the time of consult. Secondly, median follow up time was relatively short (12.4 months) with a small sample size of 58, which also did not include any patients previously treated with neoadjuvant chemotherapy or patients with PSM secondary to previously treated primary tumors, thus limiting the analytic power of this study as well as generalizability of results to a small subset of patients with primary PSM who received upfront CRS-HIPEC. Thirdly, this study does not account for the impact of adjuvant treatment regimens on the overall outcomes of patients undergoing CRS-HIPEC. With greater amounts of data obtained from further follow up, further studies can be conducted on the individual treatment outcomes tailored to peritoneal malignancies of each subtype with further subgroup analyses being performed these cases.

## 5 Conclusion

Treatment delays are predominantly contributed by healthcare-provider related factors which can be further optimized by streamlined referral processes and wider awareness towards evaluation and management of peritoneal malignancies among healthcare workers. Delayed presentation of >60 days appears to be associated with poorer disease free survival in index-presentation peritoneal surface malignancies receiving upfront CRS-HIPEC. Further studies evaluating the effects of treatment delays on survival outcomes in peritoneal malignancies would be useful in improving treatment protocols and optimizing outcomes.

## Data availability statement

The raw data supporting the conclusions of this article will be made available by the authors, without undue reservation.

## Ethics statement

The studies involving human participants were reviewed and approved by Centralized Institutional Review Board (CIRB) of Singapore Health Services, CIRB reference number 2018/2638. The patients/participants provided their written informed consent to participate in this study.

## Author contributions

JKT, JSM and CSC contributed to conception and design of the study. JKT, JCO and JSM organized the database. JKT and CL performed the statistical analysis. JKT and JSM wrote the first draft of the manuscript. All authors contributed to manuscript revision, read, and approved the submitted version.
